# Admiration and motivation: key factors in managing PTSD among firefighters

**DOI:** 10.1186/s12889-024-19420-x

**Published:** 2024-07-14

**Authors:** Yang Pan

**Affiliations:** School of Physical Education, Shandong University, Jinan, China

**Keywords:** Post-traumatic stress disorder, PTSD, Admiration, Internal motivation, External motivation

## Abstract

Post-traumatic stress disorder (PTSD) presents a significant challenge for firefighters. While research suggests that admiration may have a positive impact on individual psychological well-being, its specific influence on PTSD among firefighters remains unexplored. This study aims to investigate the association between admiration, motivation (both internal and external), and PTSD in a sample of 2156 firefighters in order to identify effective coping strategies for managing PTSD in this population. The findings indicate a statistically significant inverse relationship between admiration and PTSD, with motivation acting as a mediator. Furthermore, it is noteworthy that internal motivation is negatively correlated with PTSD in the model, while external motivation shows a positive correlation. The results suggest that feelings of admiration are associated with firefighter PTSD via motivation. Specifically, internal motivation stemming from admiration appears to have a mitigating effect on PTSD symptoms, while external motivation is linked to exacerbation of these symptoms. These results have implications for the development of theoretical frameworks and practical interventions aimed at preventing PTSD among firefighters.

## Introduction

### PTSD of firefighters

Firefighters are at an increased risk of experiencing post-traumatic stress disorder (PTSD) compared to individuals in other professions due to the inherent dangers and frequent exposure to traumatic events they encounter in the line of duty, resulting in a higher likelihood of physical injury and life-threatening situations [1–3]. From a physiological standpoint, PTSD disrupts the executive control functions of the firefighter’s brain and elicits heightened responses to threatening stimuli [4]. Psychologically, firefighters afflicted with PTSD are at an increased risk for mental health issues like depression and job burnout, as well as self-injury and suicide [5, 6]. Given the significant detrimental effects of PTSD on firefighters, it is imperative to prioritize the identification of related influencing factors and the development of strategies aimed at mitigating PTSD symptoms in order to enhance the overall well-being and effectiveness of firefighters in their demanding profession.

### The relationship between admiration and PTSD

Admiration is a distinct human emotion characterized by a deep affection and respect towards individuals who exhibit exceptional qualities or achievements [7], resulting in a positive emotional response upon witnessing their exemplary behavior or extraordinary talents [8, 9]. Meng-Lewis et al. conducted a qualitative study which revealed that the experience of admiration elicits positive emotions in individuals, leading to optimistic thoughts about their own lives and a heightened motivation to pursue personal growth and new opportunities [10]. These positive emotions and cognitive processes have been identified as potentially beneficial in coping with the risk of developing PTSD following traumatic events [11]. Contractor et al. further emphasized the significance of positive internal experiences as a crucial aspect of PTSD treatment, drawing from multiple research studies to support this assertion [12]. The aforementioned information suggests that admiration could potentially play a significant role in the prevention and mitigation of PTSD. Nevertheless, there is a lack of empirical research investigating the correlation between admiration and PTSD, prompting the development of the current study to address this gap in the literature. Therefore, research hypothesis 1 was proposed: There is a negative association between admiration and PTSD.

### The mediating role of motivation in the relationship between admiration and PTSD

Elevated levels of motivation are viewed as a mitigating factor against the development of PTSD, while diminished levels of motivation may heighten vulnerability to the disorder [13]. Currently, numerous academic studies have demonstrated that admiration possesses distinctive motivational qualities and can yield various psychological and behavioral advantages for individuals [8]. These benefits include fostering heightened attention towards the admired object, thereby motivating individuals to strive for self-improvement and diligent effort in pursuit of proximity to the admired entity [14–16]. According to the aforementioned findings, there is a close relationship between admiration and the enhancement of motivation, which in turn has a beneficial impact on the prevention of PTSD. Therefore, it can be posited that admiration may exert an influence on PTSD through its effects on individual motivation. Based on the above, research hypothesis 2 was proposed: Motivation serves as a mediating factor in the association between admiration and PTSD.

The differentiation between internal and external motivation is a fundamental concept in self-determination theory [17], delineating varying reasons or objectives driving an individual’s actions. Intrinsic motivation pertains to the drive to participate in an activity stemming from personal interest and the inherent enjoyment derived from the activity itself, whereas extrinsic motivation involves engaging in an activity with the aim of attaining external outcomes that may result from said activity [18, 19]. Given the potential disparity between internal and external motivation in influencing both inner experience and behavioral performance [19], this study seeks to investigate the mediating effects of intrinsic and extrinsic motivation on the association between admiration and PTSD among firefighters. This exploration aims to enhance our understanding of the interplay among motivation, admiration, and PTSD.

### The present study

In summary, the prevalence of PTSD among firefighters has emerged as a significant area of concern. While existing research has confirmed the beneficial impact of admiration on an individual’s psychological well-being and the importance of motivation in mitigating PTSD, the interplay between admiration, motivation, and PTSD remains unexplored. Specifically, the extent to which admiration and motivation influence the development and manifestation of PTSD among firefighters has yet to be investigated. The primary objective of this research is to investigate the correlation between admiration and PTSD among firefighters, while also examining the potential mediating influence of motivation (internal and external) on this association. The ultimate goal is to offer theoretical insights and practical recommendations for addressing PTSD issues in firefighters.

## Materials and methods

### Participants and procedure

This study selected 22 fire brigades and 130 fire stations in Tianjin as sample units and used cluster sampling to collect data on male firefighters by contacting each fire brigade commander. Approval for this research was obtained from the University Ethics Committee under Approval No. 2022-R-99. Prior to survey participation, all potential respondents were provided with the opportunity to opt in or out of the study and were briefed on its objectives. Out of 2227 questionnaires distributed, 71 were deemed ineligible due to missing data, resulting in an effective response rate of 96.81%. The final sample consisted of 2156 male firefighters, ranging in age from 18 to 50 years, with a mean age of 26.940 years (*SD* = 4.474). Their length of service ranged from six months to 30 years, with an average of 5.924 years (*SD* = 4.369). (See Table 1).

**Table 1:** Demographic information of the sample

	*N*	MIN	MAX	M	SD
Age	2156	18	50	26.940	4.474
Length of service (years)	2156	0.5	30	5.924	4.369

### Measures

#### Admiration questionnaire

Episodic recall was first used, in which participants were asked to recall the relevant scenario according to the instructions and to describe the recalled scenario in as much detail as possible to activate the admiration emotion of the participants. The specific guidance is: “A sense of admiration is the emotion and feeling triggered by someone who has an excellent ability/virtue, usually one that the individual can understand or is possible to achieve. Please describe in as much detail as possible a person who you admire in terms of abilities/virtues and achievements/virtues that you admire, as well as any abilities/virtues or abilities/virtues that you admire”. After inspiring the admiration emotion, the admiration scale developed by Chen Qing was used to measure the admiration feeling of the firefighters [20]. The scale consists of 12 items. It is scored by 5-point Likert scale, and the higher the total score of the scale, the higher the admiration level of the adolescent athlete to the object of his admiration. In this study, the Cronbach’s alpha coefficient of the scale was 0.993.

#### Work preference inventory

Firefighters’ work motivation was measured using the Chinese version of the Work Preference Inventory. The scale was revised by Wang and Chang [21] based on the Work Preference Inventory (WPI) developed by Amabile et al. [18]. The scale consists of 12 items, of which 6 items measure internal motivation and 6 items measure external motivation. The scale is based on a 5-point Likert scale (from 1 “strongly disagree” to 5 “strongly agree”), with higher scores being associated with higher work motivation. Previous studies have shown that this questionnaire has high reliability and validity among Chinese people [21]. In this study, the Cronbach’s alpha coefficient was 0.960 for the internal motivation subscale,0.921 for the external motivation subscale, and 0.964 for the total scale.

#### Impact of event scale-revised

Participants’ PTSD symptoms were measured using Impact of Event Scale-Revised (IES-R). IES-R has 22 items, using Likert 5 and scoring criteria, each item is rated on a scale of 0 (none) to 4 (extremely heavy). The total score is between 0 and 88, with higher scores indicating more severe PTSD symptoms. The Chinese version of IES-R has been verified to have a good reliability and validity level [22]. In the study, Cronbach’s alpha coefficient was 0.983.

### Statistical analysis

**Fig. 1:**
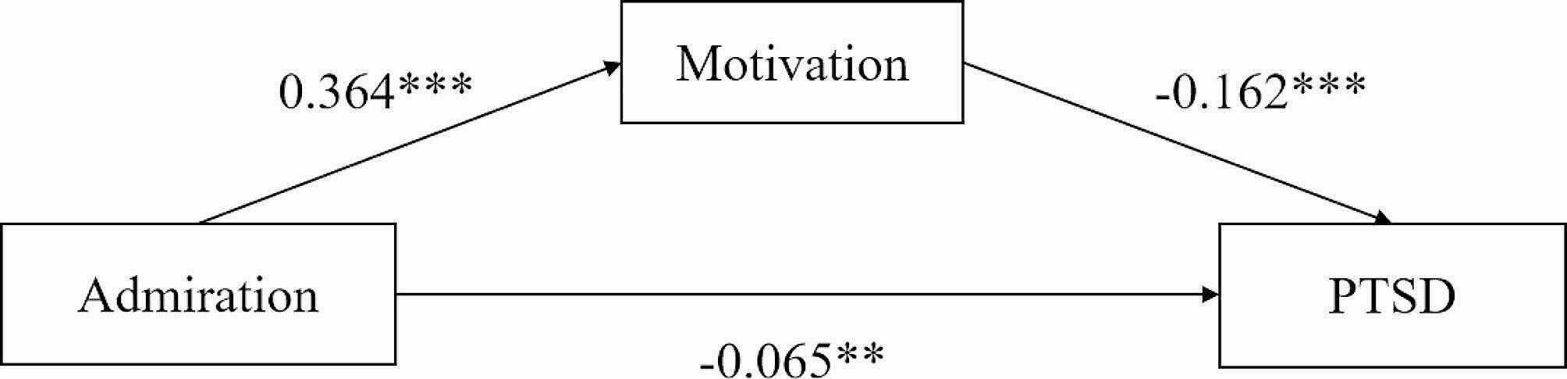
Mediation effect analysis of motivation between admiration and PTSD in firefighters. ** *P* < 0.01; *** *P* < 0.001

**Fig. 2:**
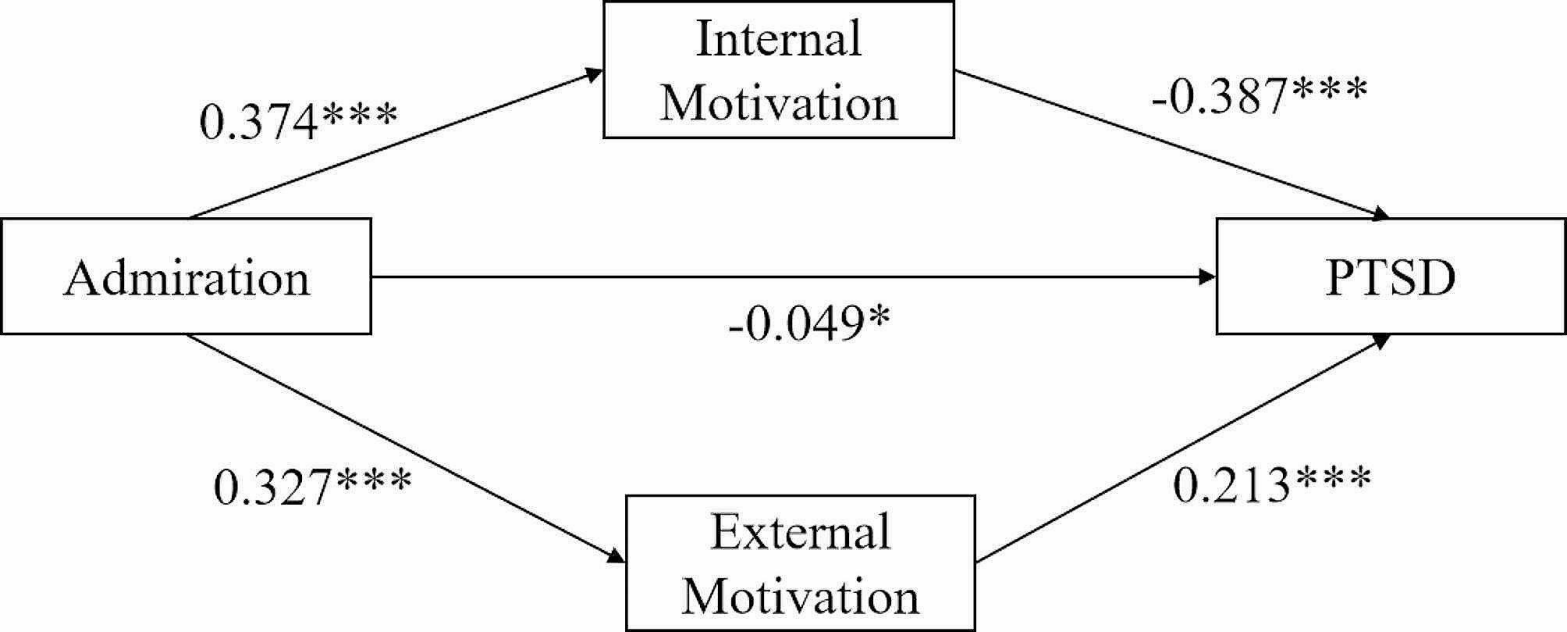
Mediation effect analysis of internal and external motivation between admiration and PTSD in firefighters. * *P* < 0.05; *** *P* < 0.001

The statistical software SPSS 24.0 was utilized to conduct statistical analysis in this study. Descriptive statistics were employed to characterize the demographic profile of a sample consisting of 2156 male firefighters, focusing on the variables of age and years of service. Furthermore, descriptive statistics were applied to determine the mean and standard deviation of each study variable (admiration, motivation, internal motivation, external motivation, PTSD) in order to provide a comprehensive overview of their distribution. Pearson correlation analysis was utilized to examine the relationship between admiration, motivation, internal motivation, external motivation, and PTSD. Upon establishing significant correlations between the variables, regression analysis was conducted using the Hayes PROCESS macro (version 3.0) to assess the model (see Figs. 1 and 2) and investigate the association between admiration and PTSD, as well as the potential mediating role of motivation (internal and external) in this relationship. In Model 1, admiration was designated as the independent variable, motivation as the mediating variable, and PTSD as the dependent variable. In accordance with the framework of Model 1, Model 2 further delineates the mediating factors as two parallel mediators, namely internal motivation and external motivation. Prior to their inclusion in the mediation analysis, each variable underwent standardization. The effects were calculated utilizing the PROCESS procedure with 5000 bootstrap samples, resulting in the generation of a 95% bias-corrected confidence interval (CI). As the CI did not encompass zero, the observed effect was deemed statistically significant at the predetermined level of α = 0.05.

## Results

### Common method bias testing

The Harman single factor test found 4 factors with eigenvalues greater than 1, but the first factor’s covariance ratio was below the 40% threshold (39.878%), indicating no major issues with common method bias in the study [23].

### Descriptive statistics and correlation analysis

Admiration, total score of motivation, internal motivation, external motivation, and PTSD mean values, standard deviations, and correlation matrices are displayed in Table 2. Admiration had a positive relationship with firefighters’ total score of motivation, internal motivation and external motivation, and a negative relationship with firefighters’ PTSD. Total score of motivation was positively correlated with firefighters’ internal motivation and external motivation, and negatively correlated with firefighters’ PTSD. Internal motivation and External motivation were negatively connected with PTSD.

**Table 2:** Descriptive statistics and correlation matrix

	1	2	3	4	5
1. Admiration	1				
2. Motivation	0.364**	1			
3. Internal Motivation	0.374**	0.962**	1		
4. External Motivation	0.326**	0.962**	0.849**	1	
5. PTSD	-0.124**	-0.185**	-0.225**	-0.132**	1
*M*	47.176	46.369	23.892	22.477	29.795
*SD*	14.184	11.847	6.162	6.158	13.957

** *P* < 0.01

### Simple mediation model analysis

#### Regression analysis with a simple mediation model

Table 3 presents the regression coefficients of the model constructed with admiration as the independent variable, PTSD as the dependent variable, and motivation as the mediating variable.

**Table 3:** The regression analysis of admiration, motivation, and PTSD in firefighters

Outcome variables	predictive variables	Goodness-of-fit indices			Regression coefficient and significance	
		*R*	*R* ^2^	F	β	t
PTSD		0.124	0.015	33.597***		
	Admiration				-0.124	-5.796***
Motivation		0.364	0.133	329.454***		
	Admiration				0.364	18.151***
PTSD		0.195	0.038	42.556***		
	Admiration				-0.065	-2.866**
	Motivation				-0.162	-7.123***

** *P* < 0.01; *** *P* < 0.001

The results indicate a statistically significant regression coefficient (*β* = -0.124, *P* < 0.001) in the association between admiration and PTSD. Furthermore, subsequent to the inclusion of motivation as a mediating variable, it was observed that admiration exhibited a statistically significant negative regression coefficient on PTSD (*β* = -0.065, *P* < 0.01), a statistically significant positive regression coefficient on firefighter motivation (*β* = 0.364, *P* < 0.001), and a statistically significant negative regression coefficient between motivation and PTSD (*β* = -0.162, *P* < 0.001). The regression weights for the path analysis can be visualized in Fig. 1.

#### Mediation analysis of the simple mediation model

Table 4 displays the utilization of the bias-corrected 5000 sample bootstrap method to estimate the total, direct, and indirect effects within the model. The findings indicate that the total effect size of the model was statistically significant (total effect = -0.124; 95%CI: -0.166, -0.082). Specifically, admiration exhibited a significant direct effect on PTSD (direct effect = -0.065; 95%CI: -0.110, -0.021), while motivation was found to play a significant mediating role between admiration and PTSD (indirect effect = -0.059; 95%CI: -0.077, -0.042). The above results suggest that motivation partially mediates the relationship between admiration and PTSD in firefighters.

**Table 4:** The mediation analysis of motivation between admiration and PTSD in firefighters

Effect	Standardized Coefficient	Bootstrap SE	Bootstrap 95%CI Lower Limit	Bootstrap 95%CI Upper Limit
Total effect	-0.124	0.021	-0.166	-0.082
Direct effect	-0.065	0.023	-0.110	-0.021
Indirect effect	-0.059	0.009	-0.077	-0.042

### Parallel mediation model analysis

#### Regression analysis with a parallel mediation model

Table 5 displays the regression coefficients of the model constructed with admiration as the independent variable, PTSD as the dependent variable, and internal and external motivation as parallel mediators.

**Table 5:** The regression analysis of admiration, internal motivation, external motivation, and PTSD in firefighters

Outcome variables	predictive variables	Goodness-of-fit indices			Regression coefficient and significance	
		*R*	*R* ^2^	F	β	t
Internal motivation		0.374	0.140	350.341***		
	Admiration				0.374	18.717***
External motivation		0.327	0.107	256.944***		
	Admiration				0.327	16.030***
PTSD		0.255	0.065	49.785***		
	Admiration				-0.049	-2.162*
	Internal motivation				-0.387	-9.616***
	External motivation				0.213	5.388***

* *P* < 0.05; *** *P* < 0.001

The findings indicated that admiration had a statistically significant positive effect on both intrinsic (*β* = 0.374, *P* < 0.001) and extrinsic (*β* = 0.327, *P* < 0.001) motivation among firefighters. Additionally, the regression coefficient for internal motivation in relation to PTSD was found to be negative (*β* = -0.387, *P* < 0.001), whereas the regression coefficient for external motivation in relation to PTSD was positive (*β* = 0.213, *P* < 0.001). Furthermore, the direct impact of admiration on PTSD remained statistically significant even after incorporating internal and external motivation as mediating factors (*β* = -0.049, *P* < 0.05). The regression weights for the path analysis can be observed in Fig. 2.

#### Mediation analysis of the parallel mediation model

Table 6 presents the utilization of the bias-corrected 5000 sample bootstrap method for estimating the effect size of the model. The findings indicate a statistically significant effect of admiration on PTSD (direct effect = -0.049; 95%CI: -0.093, -0.005), with motivation serving as a mediator in this relationship (total indirect effect = -0.075; 95%CI: -0.096, -0.057). More specifically, in this study, it was found that intrinsic motivation had a negative mediating effect on the relationship between admiration and PTSD, with an indirect effect of -0.145 (95%CI: -0.182, -0.113). Conversely, extrinsic motivation had a positive mediating effect on the relationship between admiration and PTSD, with a mediating effect of 0.070 (95%CI: 0.045, 0.098). The bootstrap analysis revealed that none of the 95% confidence intervals included zero, indicating a significant mediating effect of both intrinsic and extrinsic motivation on the relationship between admiration and PTSD. The results indicate that admiration may have a dual effect on PTSD, with internal motivation potentially reducing symptoms while external motivation may exacerbate them. Overall, the mediating effect of internal motivation in the relationship between admiration and PTSD is more pronounced than that of external motivation. This suggests that while admiration may exacerbate PTSD through external motivation, it also has a greater mitigating effect on PTSD through internal motivation. Thus, admiration plays a significant role in the modulation of PTSD through motivational mechanisms.

**Table 6:** The mediation analysis of internal motivation and external motivation between admiration and PTSD in firefighters

Effect	Standardized Coefficient	Bootstrap SE	Bootstrap 95%CI Lower Limit	Bootstrap 95%CI Upper Limit
Total effect	-0.124	0.021	-0.166	-0.082
Direct effect	-0.049	0.023	-0.093	-0.005
Total indirect effect	-0.075	0.010	-0.096	-0.057
AD → IM → PTSD	-0.145	0.018	-0.182	-0.113
AD →EM → PTSD	0.070	0.013	0.045	0.098

AD = admiration; IM = internal motivation; EM = external motivation

## Discussion

### The relationship between admiration and PTSD in firefighters

The findings indicated an inverse relationship between admiration and PTSD among firefighters. Those with heightened levels of admiration displayed fewer symptoms of PTSD, supporting research hypothesis 1 and aligning with existing literature suggesting that feelings of admiration can positively impact individual psychological well-being [8, 10]. The experience of admiration can significantly heighten an individual’s self-awareness and motivate them to surmount challenges and barriers in order to achieve a meaningful goal [9]. This cognitive capacity for self-awareness has been shown to facilitate the recovery process for individuals with PTSD [11, 24]. Furthermore, individuals with PTSD frequently experience psychophysiological dysfunction [25]. The social emotion of admiration encompasses complex cognitive processing and physiological regulation, impacting factors such as blood pressure, hormonal regulation, heart rate, and cerebral blood flow, leading to feelings of warmth, energy, and increased heart rate [14, 26]. Hence, firefighters who possess a heightened sense of admiration are more likely to sustain improved psychological and physiological well-being, consequently mitigating the likelihood of experiencing symptoms of PTSD. 

### The mediating role of motivation in the relationship between admiration and PTSD in firefighters

The findings of the study indicate that motivation serves as a mediator in the association between admiration and PTSD among firefighters, aligning with hypothesis 2. Specifically, the results demonstrate a positive correlation between firefighters’ feelings of admiration and their motivation. This relationship can be attributed to the tendency for individuals to emulate characteristics of admired figures, suggesting an active process rather than a passive one [8, 27]. Consequently, feelings of admiration are implicated in bolstering motivation levels. The findings of the study also indicate a negative correlation between motivation and PTSD in firefighters, aligning with Kaplan et al.’s assertion that motivation plays a significant role in the remission and onset of PTSD [13]. The aforementioned analysis suggests that a strong sense of admiration can serve as a catalyst for firefighters to proactively adopt their admired individuals as role models, exerting effort and continuously enhancing their skills to approach success. This heightened motivation enhances firefighters’ psychological ability in confronting potentially traumatic incidents at work, thereby mitigating and preventing symptoms of PTSD in this occupational group.

The primary discovery of this research indicates that internal and external motivation exhibit distinctly different effects on the correlation between admiration and PTSD. More specifically, internal motivation demonstrated a negative association with PTSD within the model, whereas the link between external motivation and PTSD displayed a positive relationship. This suggests that internal motivation, driven by feelings of admiration, contributes to the alleviation of PTSD among firefighters, whereas external motivation, also stemming from admiration, may hasten the onset of PTSD in firefighters. This discovery corroborates the notion that external motivation can yield adverse consequences [28]. Furthermore, it offers partial validation to van der Rijt’s concept of “the Vice of Admiration” [29], suggesting that admiration, when lacking in fostering self-improvement motivation, can detrimentally impact an individual’s emotional and mental well-being. However, the results also indicate that external motivation may exacerbate PTSD symptoms in firefighters, whereas internal motivation has a more effective impact in alleviating the condition. Consequently, admiration serves as a protective factor in reducing PTSD by bolstering firefighters’ motivation. These results highlight the importance of promoting internalizing motivation through admiration in interventions designed to mitigate PTSD in firefighters.

In the practical application of managing PTSD among firefighters, it is recommended to utilize lectures, training sessions, and daily public awareness campaigns focused on career-related examples to inspire admiration and guide internal motivation and psychological growth among firefighters. Meanwhile, it is imperative to provide counseling to firefighters in order to mitigate their heightened preoccupation with external evaluation resulting from direct comparisons with admired individuals, thereby preventing adverse emotions stemming from external motivation.

### Implications and limitations

This study holds significant implications. PTSD can significantly impair the physical and mental well-being, as well as job performance, of firefighters. The management of PTSD in this population has garnered increasing interest. The findings of this research offer recommendations for mitigating PTSD symptoms among firefighters and promoting their psychological states following exposure to traumatic incidents. These insights can inform the development of effective psychological interventions and counseling services for firefighters. Specifically, it is imperative to enhance the development of admiration and motivation for firefighters through consistent training and daily life practices in order to mitigate and prevent symptoms of PTSD among this population. It is important to emphasize the necessity of guiding firefighters in internalizing their work motivation in order to prevent the exacerbation of PTSD. By fostering a desire for self-improvement through admiration for specific objectives and cultivating a stronger internal motivation for work, PTSD among firefighters can be more effectively managed.

Our study is constrained by several limitations. Firstly, due to the cross-sectional nature of the questionnaire survey, it is unable to establish a definitive causal relationship between variables, potentially leading to subjective research outcomes. Secondly, the study exclusively focused on male firefighters within the Chinese cultural context, limiting the generalizability of the findings to other cultural settings or female firefighter populations. In light of the aforementioned constraints, forthcoming research endeavors may employ longitudinal tracking or experimental methodologies to quantitatively assess variables in a more objective manner, thereby yielding more robust evidence for the causal link between admiration, motivation, and PTSD. Furthermore, future investigations could enhance the generalizability of the study’s model framework by examining firefighters across various gender demographics and cultural contexts.

## Conclusion

This research investigated strategies for managing PTSD in firefighters through an examination of the relationship between admiration, motivation, and PTSD. The findings revealed that admiration was negatively associated with PTSD among firefighters. Additionally, motivation was identified as a mediator in the relationship between admiration and firefighter PTSD, with internal and external motivations exerting distinct influences. Internal motivation, stemming from a sense of admiration, was found to alleviate firefighter PTSD, whereas external motivation was linked to an exacerbation of symptoms. This study could serve as a valuable resource for informing the development of preventative measures for PTSD among firefighters.

## Data Availability

The data that support the findings of this study are available from the corresponding author upon reasonable request.
